# Effectiveness of multimedia courses in improving self-care among patients with breast cancer undergoing radiotherapy

**DOI:** 10.1186/s13014-023-02312-6

**Published:** 2023-07-11

**Authors:** Huei-Fan Yang, Wen-Wei Chang, Ying-Hsiang Chou, Jing-Yang Huang, Ya-Fang Ke, Pei-Fang Tsai, Hsiu-Man Chan, Hsueh-Ya Tsai, Hsien-Chun Tseng, Shih-Tsung Chang, Yueh-Chun Lee

**Affiliations:** 1 Department of Radiation Oncology, Chung Shan Medical University Hospital, Taichung, Taiwan; 2 Department of Nursing, Chung Shan Medical University Hospital, Taichung, Taiwan; 3 Department of Biomedical Sciences, Chung Shan Medical University, Taichung, Taiwan; 4 Department of Medical Imaging and Radiological Sciences, Chung Shan Medical University, Taichung, Taiwan; 5 School of Medicine, Chung Shan Medical University, Taichung, Taiwan; 6 Institute of Medicine, Chung Shan Medical University, Taichung, Taiwan; 7 Department of Medical Research, Chung Shan Medical University Hospital, Taichung, Taiwan

**Keywords:** Radiation therapy, Multimedia health education video, Self-care

## Abstract

**Background:**

Ninety percent of patients receiving radiation therapy experience side effects. Busy schedules and intensive health education programs may lead to incomplete education content delivery and inaccurate patient self-care implementation. This study investigated whether multimedia health education improves the accuracy of patient self-care implementation compared with paper-based education.

**Methods:**

From March 11, 2020 to February 28, 2021, 110 patients were randomly divided into experimental and control groups, each comprising 55 participants. Paper-based materials were used along with multimedia materials. Radiology self-care awareness questionnaires were administered to both groups before the first treatment and on day 10. The differences in radiology self-care awareness between the two groups was analyzed with inferential statistics, independent t tests, categorical data, and Pearson’s chi-squared test. Differences between the two groups were considered significant at a *p* value of < 0.05.

**Results:**

The treatment accuracy rate improved from 10.9 to 79.1% in the control group and from 24.8 to 98.5% in the experimental group, indicating an improvement in both groups. The difference was significant. These results indicate that the intervention could improve the effectiveness of self-care.

**Conclusions:**

Participants who used pretreatment multimedia health education exhibited a higher rate of having a correct understanding of treatment self-care than did the control group. These findings can inform the development of a patient-centered cancer treatment knowledge base for improved quality of care.

## Introduction

According to the Taiwanese National Health Administration cancer registry, breast cancer is the third leading cause of cancer-related deaths among women, and a total of 2,913 women died from breast cancer in 2021 [1]. In addition to chemotherapy, pharmacotherapy, and surgery, radiotherapy is currently among the primary treatments for breast cancer, and it is crucial for reducing recurrence rates [2]. Combined postoperative radiation therapy can enhance local tumor control, reduce the risk of posttreatment complications, and increase survival rates [3]. Skin reactions, arm lymphedema, breast tissue fibrosis, and tiredness are common side effects experienced by breast cancer patients undergoing radiation therapy. To alleviate these discomforts and prevent skin complications, it is crucial to provide self-care education prior to treatment [4]. This study was conducted with guidance from the self-regulation theory, which suggests that effective coping with an experience is closely linked to the level of preparedness individuals have for that experience. According to this theory, specific objective aspects of an experience, such as physical sensations, temporal features, environmental factors, and the causes of those sensations, play a significant role in guiding patients' coping strategies and minimizing distress and disruptions in their daily lives [5, 6]. Indeed, Abdollahi et al. [7] have demonstrated that self-care behaviors positively predicted the quality of life and resilience in breast cancer patients. When undergoing an unfamiliar treatment or facing an unfamiliar environment, 90% of patients experience anxiety and often ignore self-care instructions provided by their health care team [8–10]. Patient self-care education and information regarding radiotherapy side effects are provided prior to treatment so that patients can fully understand related processes and psychologically prepare themselves; this can reduce their anxiety levels and fear of the unknown [11]. The uncertainty and common side effects of treatment tend to make patients feel anxious and uneasy; therefore, self-care health education content is easier for patients to understand when it is delivered through everyday speech or through comprehensible text and images [12]. In this era of changing medical environments and advanced technology, the use of multimedia and the scanning of quick response (QR) codes provide patients and their families with more diverse and effective care guidance models. Some challenges to the implementation of patient self-care include the provision of radiation therapy in outpatient settings, a short treatment duration, the unsuitable psychological and physical status of some patients, and the lack of time available for health education [9, 13]. In this context, educational media tools may be an appropriate means of providing patient education because, when properly selected, these tools can enhance learner motivation and deep learning in addition to saving time [11]. Numerous studies have reported that the integration of nursing instruction with multimedia teaching models is more effective at improving patient self-care awareness compared with the use of nursing instruction leaflets or manuals alone [14]. Multimedia teaching breaks the monotony of print and text; it allows learners to rapidly achieve learning objectives, shortens learning time regardless of location, and ensures that teaching materials can be updated at any time; therefore, it provides greater freedom and flexibility [15–18]. These advantages of multimedia nursing guidance enhance patients’ visual and auditory learning. Visual and audio stimulation can enhance patient concentration during the learning experience, making it easier for patients to understand complex issues in clinical settings [19–21]. In addition to health education leaflets and instructional manuals, multimedia health education should be incorporated into routine clinical teaching models to improve patients’ quality of care and knowledge absorption [22, 23]. For example, Kuo et al. [24] have reported that using multimedia health education intervention could reduce the anxiety of people who went for mammography screening of breast cancer, but such intervention did not relief the pain during mammography. Shih et al. demonstrated that the combination of multimedia with traditional routine education among pre-operative breast cancer patients displayed partial effects on anxiety reduction [25]. However, the effectiveness of multimedia-based self-care education for breast cancer patients who received radiotherapy remains to be explored.

Due to nursing staff shortages and limited time for health education, patients face inadequate nursing guidance, resulting in challenges in understanding and adhering to self-care instructions. This leads to unclear perceptions of self-care, inaccurate execution of tasks, and heightened acute side effects, causing anxiety and an increased rate of treatment interruptions. This study aims to assess the effectiveness of audio-visual models and paper-based education leaflets in promoting self-care among breast cancer patients receiving radiation therapy. The objective is to determine if these tools can provide valuable information and support, leading to positive self-care behaviors.

## Method

### Study design

This study was approved by the Ethics Committee for Human Subjects (IRB) of the relevant Chung Shan Medical University Hospital with an approval number of 20,005. All the enrolled participants were provided verbal and written information regarding the study and provided written informed consent. Random assignment was conducted using the random assignment software provided by iClinical, developed by the Office of Data Science, Taipei Medical University (http://biostat.tmu.edu.tw/iclinical/). The study included an experimental group and a control group. The experimental group received multimedia education, while the control group used traditional paper-based education.

### Research site and participants

Participants were recruited from the department of radiation oncology of a medical center in central Taiwan. All participants were (a) patients with breast cancer undergoing radiation therapy for the first time, (b) patients with clear consciousness and the ability to understand Mandarin Chinese, and (c) patients who were willing to respond to the questionnaire after the research process and purpose were explained to them. The following participants were excluded: (a) patients who had previously undergone this treatment and (b) patients with reduced mental capacity or the ability to satisfactorily participate in this study.

A total of 110 participants were randomly assigned to the experimental group and the control group. Each group contained 55 participants. A posttest questionnaire was administered after 2 weeks of treatment. The data collection period was from March 11, 2020 to February 28, 2021.

### Research process

With the simulated photography day being the first day of self-care education, radiotherapy self-care education was provided. After 2 weeks of treatment, the effectiveness levels of the first and second health education sessions were evaluated. Nursing education was reinforced to address any uncertainties. During the treatment period, the patients had the opportunity to enquire about the content and methods of self-care health education at outpatient clinics once a week; these sessions were used to investigate the degree to which patients absorbed health and self-care knowledge.

### Research tools

To our knowledge, this topic has not been investigated in Taiwan; therefore, no preexisting scale could be appropriately used. The questionnaire was developed with reference to previous studies, the research purpose, expert advice, and the content of radiotherapy self-care education. The questionnaire items were divided into the following sections: (a) basic information (b), satisfaction with the radiotherapy self-care multimedia textbooks, (c) satisfaction with the radiotherapy self-care health education leaflet, (d) satisfaction with the health education of nursing educators, and (f) a radiotherapy self-care assessment.

### Data collection and analysis

Two data collection stages were employed. The first stage involved preliminary research to (1) explore potential problems in implementing the research tools, (2) test the feasibility of the research process, and (3) revise the research tools in accordance with our findings. Prior to testing, the multimedia video, health education leaflet, and questionnaire content were all finalized for expert validity. The second stage was a formal test. Statistical analysis of the collected data was conducted using SPSS 19.0 (IBM, Armonk, NY, USA). Descriptive statistics were used to analyze numbers, percentages, means, and standard deviations (SDs) in order to assess the participants’ comprehension of radiotherapy self-care. Inferential statistics, independent *t* tests (unpaired *t* test), categorical data, and Pearson’s chi-squared test were used to analyze the differences between patients’ comprehension of radiotherapy-related self-care multimedia materials and health education leaflets. Differences between the two groups were considered significant at a *p* value of < 0.05.

### Reliability and validity

A content validity index (CVI) was used to test the content validity of the questionnaire. Content validity testing was conducted by five scholars and clinical experts in related fields, including a nurse with more than 10 years of clinical experience and a relevant specialist qualification and physicians and radiologists with more than 10 years of experience in the field of clinical tumor radiology. A four-point scoring method was adopted. After a review, the CVI was approved and used by the experts. The sum of the scores greater than or equal to three was divided by the number of all experts to determine the consistency of questionnaire items.

### Textbook content

The breast cancer radiotherapy self-care teaching leaflet and self-care teaching video were designed by the research team and colleagues such that they were integrated with a previous breast cancer radiotherapy self-care education leaflet; the content was designed to minimize the use of medical jargon and enhance patient comprehension. In addition to the written leaflet, the team members used Microsoft PowerPoint and audio recordings to produce an educational video based on the content of the original self-care education leaflet. The topics covered were radiotherapy instructions, common side effects, and the principles of self-care. The health education leaflets and multimedia presentation were prepared for education sessions at the research site.

### Ethical considerations

This study was approved by the Ethics Committee for Human Subjects (IRB) of the relevant Chung Shan Medical University Hospital (No. 20005). The purpose of the study and the process of data collection were explained to eligible patients. Patients then conveyed verbal acceptance and signed a written consent form. Participants were allowed to terminate their participation in the study at any time during the study period if they so wished. Neither participation nor refusal to participate would affect the quality of their medical care. Anonymous coding was used to protect patient privacy, and this study was not sponsored by any suppliers.

## Results

### Reliability analysis

For each scale in this study, the reliability coefficient was between 0.7 and 0.9, indicating a high level of reliability. The Cronbach’s alpha results are as follows: satisfaction with the health education leaflet, 0.79; satisfaction with the health education video, 0.94; and satisfaction with nurse-led education at the radiation oncology department, 0.82.

### Validity analysis

The first part of questionnaire, which examined satisfaction with the health education leaflet, had a total of 10 questions, and the average CVI score was 0.975. The second part of the questionnaire, which covered satisfaction with video-based education, had a total of nine questions, and the average CVI score was 0.972. The third part of the questionnaire, which was a self-care comprehension assessment, had a total of six questions, and the average CVI score was 0.979. The questionnaire was thus valid.

### Participant characteristics

The sample comprised 110 participants. Both groups had only women. The age distribution ranged from 32 to 78 years. The average age of those in the experimental group was 53.78 years (SD = 10.16), and the average age of those in the control group was 56.16 years (SD = 11.43). In terms of marital status, the largest proportion of participants were married. In terms of residential status, the largest proportion of participants lived with their spouses and children. No statistically significant differences were observed between the two groups (*p* > 0.05; Table 1).

**Table 1:** Demographic and clinical characteristics in health education leaflet group and audiovisual education group

Variable	Health education leaflet (n = 55)	Audiovisual health education (n = 55)	*p*-value
Age (year), mean ± SD	56.16 $\pm \hspace{0.17em}$11.43	53.78 $\pm \hspace{0.17em}$10.16	0.2505
Education level			0.1793
High school and below	34 (61.82%)	27 (49.09%)	
College, university and above	21 (38.18%)	28 (50.91%)	
Profession			0.1799
Have a job	29 (52.73%)	37 (67.27%)	
Housekeeping	9 (16.36%)	9 (16.36%)	
Retire	17 (30.91%)	9 (16.36%)	
BMI, mean ± SD	24.24 $\pm \hspace{0.17em}$3.40	24.60 $\pm \hspace{0.17em}$2.89	0.5454
18–24	33 (60.00%)	15 (27.27%)	
24–27	14 (25.45%)	33 (60.00%)	
> 27	8 (14.55%)	7 (12.73%)	
Cancer clinical staging			0.1695
IA, IB	18 (32.73%)	23 (41.82%)	
IIA, IIB	12 (21.82%)	10 (18.18%)	
IIIA, IIIB	15 (27.27%)	19 (34.55%)	
IV	10 (18.18%)	3 (5.45%)	
Laterality of breast cancer			0.5162
Left	42 (76.36%)	39 (70.91%)	
Right	13 (23.64%)	16 (29.09%)	
Surgery treatment	55 (100%)	55 (100%)	1.0000
Total radiation dose			0.9166
≤ 50.5	8 (14.55%)	8 (14.55%)	
50.5–55	21 (38.18%)	19 (34.55%)	
> 55	26 (47.27%)	28 (50.91%)	
Number of radiation			0.3066
≤ 20	1 (1.82%)	4 (7.27%)	
21–25	28 (50.91%)	23 (41.82%)	
> 25	26 (47.27%)	28 (50.91%)	

### Radiotherapy awareness and exposure

**Fig. 1:**
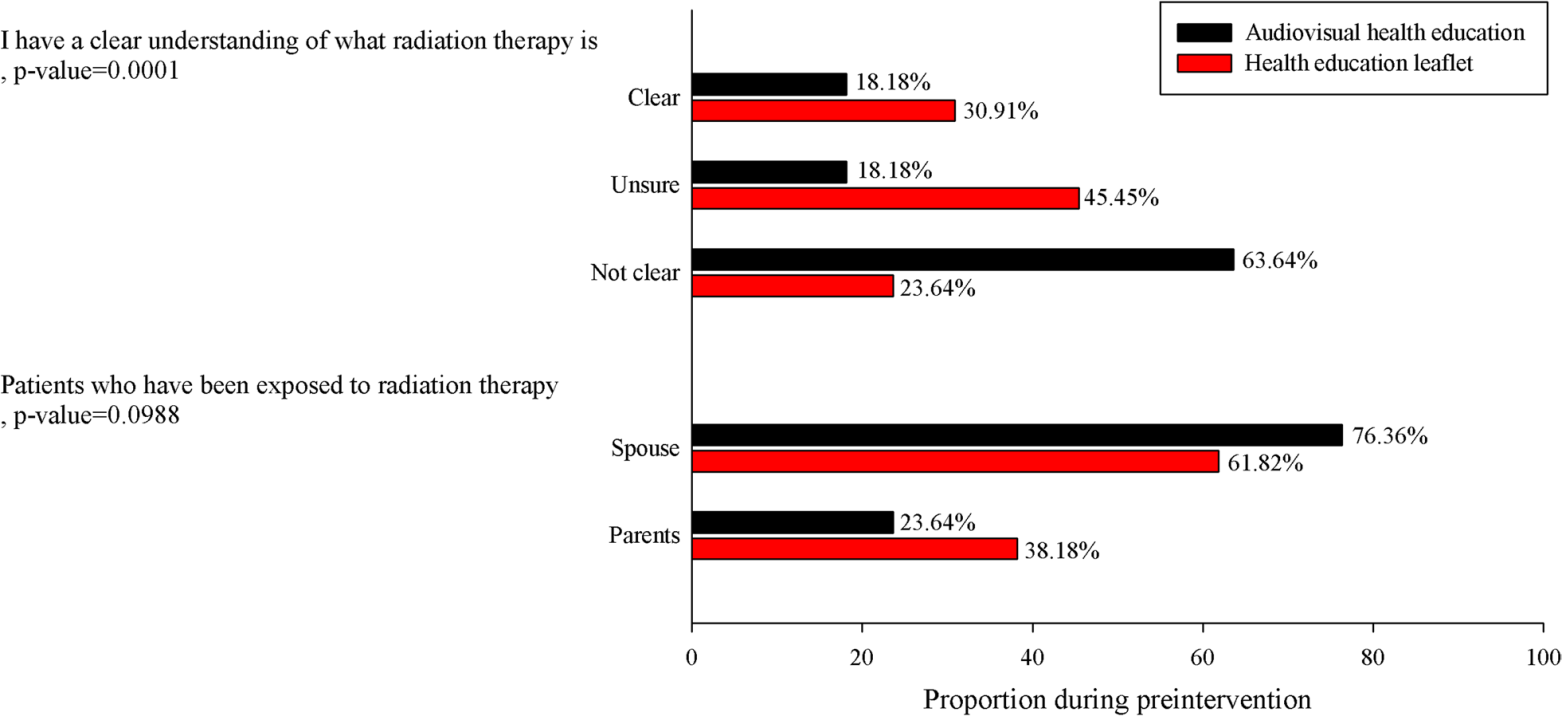
Preintervention questionnaire results

Before the health education intervention, differences in the understanding of and exposure to radiotherapy were assessed in the two groups. As indicated in Fig. 1, a significant difference was noted between the experimental group and control group regarding participants’ understanding of what radiotherapy is (*p* = 0.0001).

### Satisfaction after the two health education interventions

**Fig. 2:**
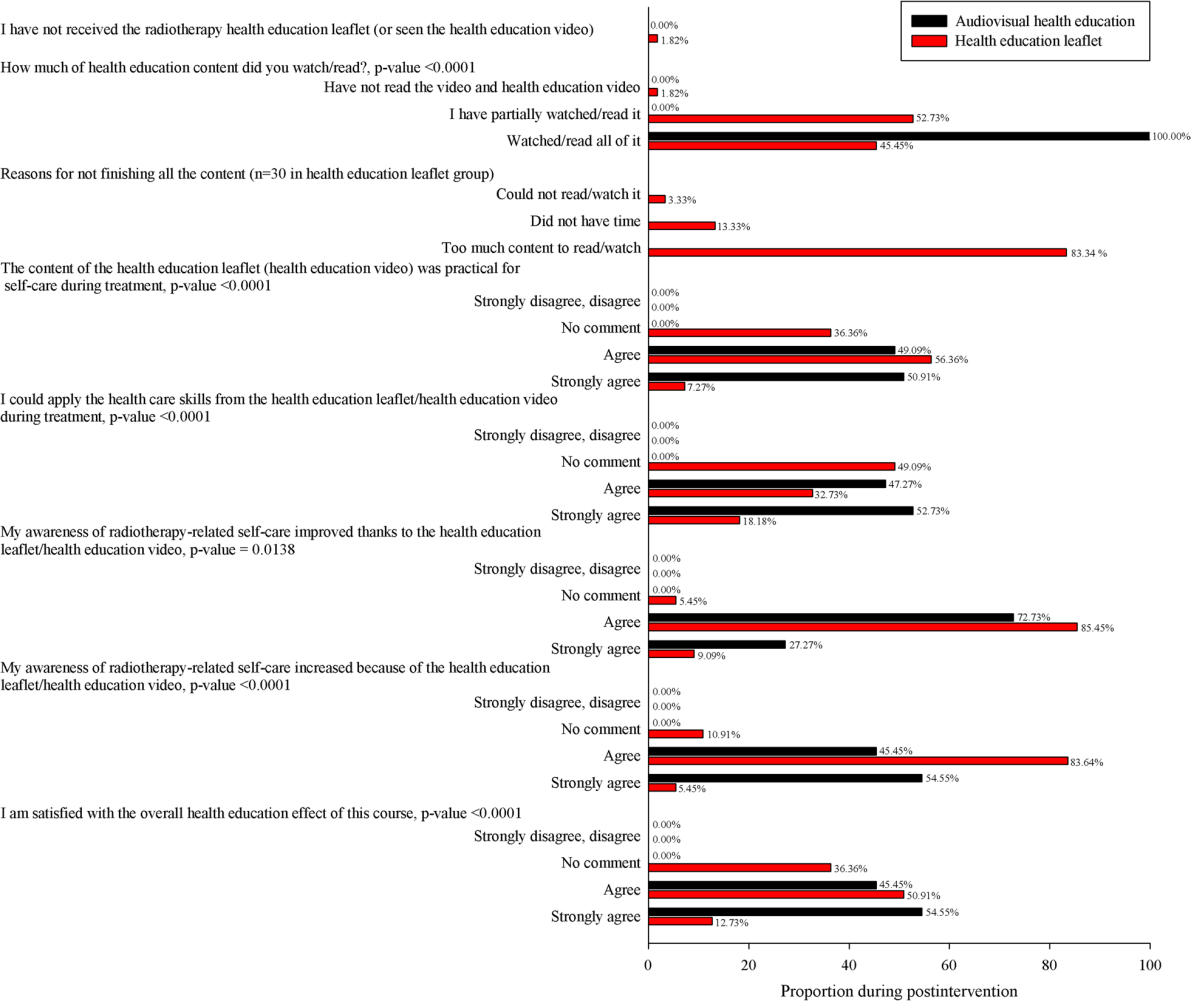
Postintervention questionnaire results

The two groups were administered questionnaires before and after the interventions, and posttests were administered 2 weeks after treatment. Differences between the groups were analyzed. The experimental group returned 55 responses (100%), and the control group returned 25 responses (45.45%; *p* < 0.001). When asked whether the content was practical for self-care during treatment, 55 people (100%) in the experimental group agreed or strongly agreed, and 35 people (63.56%) in the control group expressed such agreement (*p* < 0.001). With regard to Professionalism Satisfaction, 55 people (100%) in the experimental group agreed or strongly agreed, and 28 people (50.91%) in the control group agreed (*p* < 0.001). With regard to content professional satisfaction, 55 people (100%) in the experimental group agreed or strongly agreed, and 52 people in the control group expressed such agreement (94.54%). On the statement “I increased my awareness of radiation self-care using the health education leaflet or health education video,” 55 people in the experimental group agreed or strongly agreed (100%), and 49 people in the control group expressed such agreement (89.09%). With regard to satisfaction with the overall effect of health education, 55 people (100%) in the experimental group agreed or strongly agreed, and 35 people (63.64%) in the control group expressed such agreement (*p* < 0.001; Fig. 2).

## Discussion

With the current health care burden and the shortage of nursing personnel, nursing staff are unable to allocate sufficient time to instruct patients. Only limited nursing guidance is feasible in current clinical settings. Accurate radiotherapy-related self-care education is a key duty of nursing staff. The research findings were as expected in light of the well-known advantages of multimedia tools for education. These include the capability to observe progress, increased learning attractiveness, and good usability for patients with a low literacy level [20, 21].

Significant between-group differences were observed in patient comprehension. The results indicated that the comprehension of the control group increased from 10.9 to 79.1% and the comprehension of the experimental group increased from 24.8 to 98.5% (*p* < 0.001; Table 2). The differences between the groups were significant. This result is consistent with a previous report by Kao et al. [26], which utilized multimedia teaching to improve the accuracy rate of intermittent catheterization among primary caregivers. Another study by Huang et al. [27] demonstrated that multimedia health education significantly enhanced insulin self-injection skills in patients with type 2 diabetes. In a clinical trial evaluating the effectiveness of a multimedia education tool in lung cancer patients, those who received multimedia education through an iPad application reported significantly higher satisfaction scores compared to those who received standard education. This included a higher willingness to recommend the educational materials to others [28]. Collectively, these studies, along with our data, suggest that multimedia education tools are valuable in improving the quality of care for cancer patients.

**Table 2:** Pretest and posttest comprehension assessment results

Variable	Health education leaflet (n = 55)	Audiovisual health education (n = 55)	*p*-value
Pretest	10.91 ± 13.30	24.84 ± 23.54	< 0.0001
Post test	79 ± 17.32	98 ± 6.63	< 0.0001

Analyses of each item indicated differences in educational level, marital status, and residence status; however, some of these differences were nonsignificant. Patients with only an elementary school education exhibited lower disease-related self-care knowledge than did those with a higher education level. Regarding marriage and residence status, unmarried, divorced, widowed, and single patients exhibited higher disease-related self-care knowledge. This finding may be ascribed to patients needing to independently deal with both serious and trivial matters and patients without a spouse needing to pay greater attention to their personal affairs. Many findings cannot be adequately explained; further research is required to investigate cause and effect. For questions 4, 5, and 6, the multimedia textbook group had a higher correct answer rate in the pretest and posttest. The control group reported lower correct answer rates than did the experimental group (Table 3). This may be a result of patients not fully reading the health education leaflet. For future interventions, each major item of the health education leaflet should be retyped, highlighted in bold, and accompanied by detailed descriptions. This may encourage patients to read the entire leaflet.

**Table 3:** Radiotherapy self-care assessment

Variable	Health education leaflet (n = 55)	Audiovisual health education (n = 55)	*p*-value
1. Radiation therapy is administered 5 days a week			0.3190
Incorrect before and incorrect after	0 (0.00)	0 (0.00)	
Incorrect before and correct after	38 (69.09)	33 (60.00)	
Correct in both pretest and posttest	17 (30.91)	22 (40.00)	
2. Radiation therapy has no side effects			0.4598
Incorrect before and incorrect after	3 (5.45)	1 (1.82)	
Incorrect before and correct after	38 (69.09)	36 (65.45)	
Correct in both pretest and posttest	14 (25.45)	18 (32.73)	
3. I should take care to cool and moisturize my skin during treatment			0.0114
Incorrect before and incorrect after	2 (3.64)	0 (0.00)	
Incorrect before and correct after	50 (90.91)	42 (76.36)	
Correct in both pretest and posttest	3 (5.45)	13 (23.64)	
4. During treatment, a balanced diet should contain high-quality protein-rich foods			0.0215
Incorrect before and incorrect after	5 (9.09)	0 (0.00)	
Incorrect before and correct after	49 (89.09)	50 (90.91)	
Correct in both pretest and posttest	1 (1.82)	5 (9.09)	
5. A physician must assess responses to treatment weekly			< 0.0001
Incorrect before and incorrect after	28 (50.91)	2 (3.64)	
Incorrect before and correct after	27 (49.09)	47 (85.45)	
Correct in both pretest and posttest	0 (0.00)	6 (10.91)	
6. I can interrupt treatment or rest on my own if I feel uncomfortable during treatment			< 0.0001
Incorrect before and incorrect after	31 (56.36)	2 (36.40)	
Incorrect before and correct after	23 (41.82)	35 (63.64)	
Correct in both pretest and posttest	1 (1.82)	18 (32.73)	

**Fig. 3:**
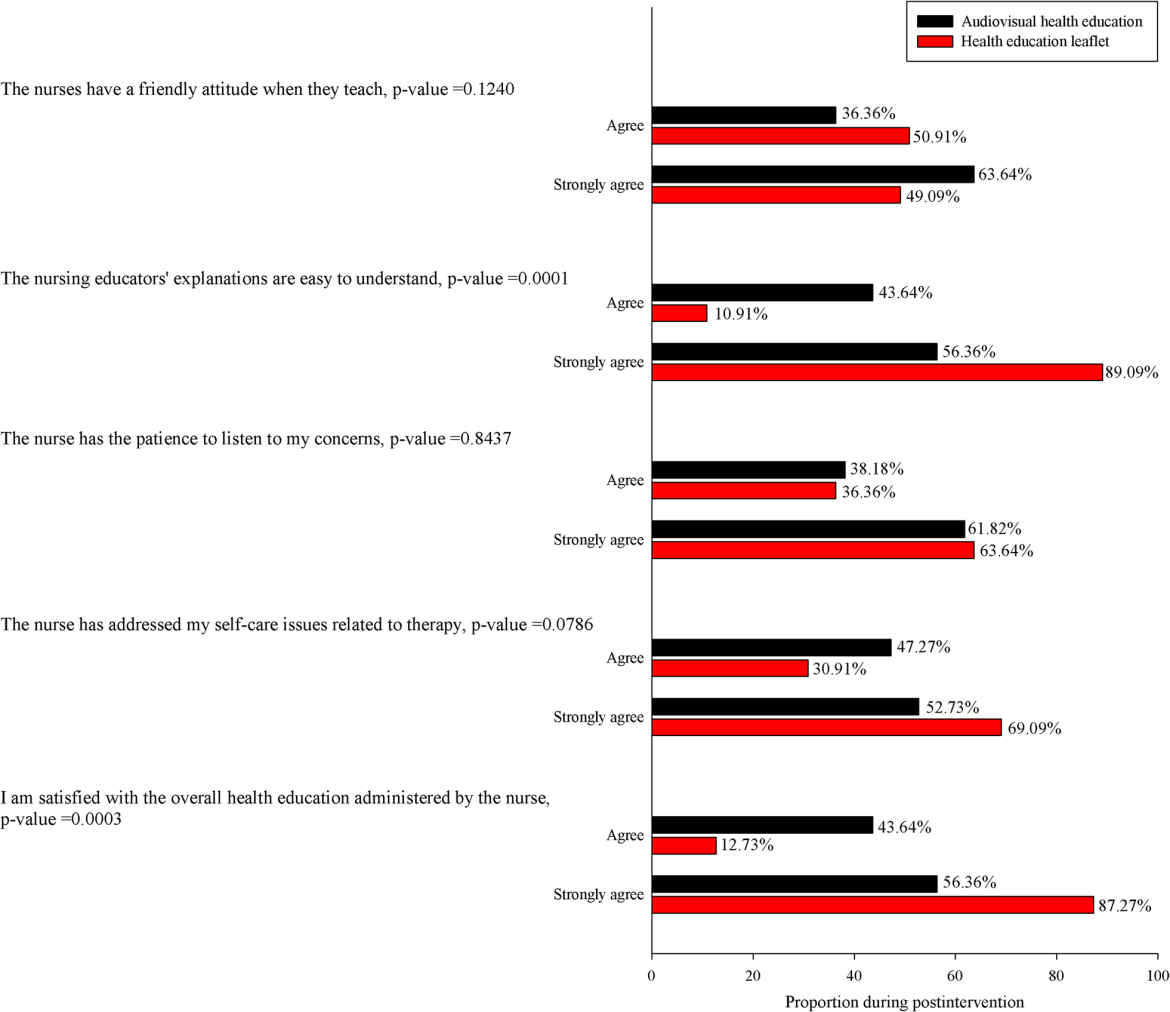
Satisfaction with the health education of nursing educators

Although multimedia videos and recordings can improve the effectiveness of health education, they cannot completely replace psychological support and comfort with patients in this study were satisfied with the general health education provided by nurses. In total, 99% of respondents from the two groups agreed that the nurses had a friendly attitude when they taught; thus, they indicated acceptance of traditional health education. This could explain why the leaflet group reported higher levels of agreement than the multimedia group for the statement “The nurse practitioner was patient and listened to my concerns.” (Fig. 3).

Multimedia should be incorporated into routine clinical processes to help patients with cancer improve their self-care ability and recover smoothly, to improve patient satisfaction with the quality of clinical care, and to establish a win–win effect between nursing and disease. Different health education models should be applied for the convenience of patients from different ethnic groups.

### Limitations of this study

The study is subject to several limitations. Firstly, the data used in the study is obtained from a single institution, which may restrict the generalizability of the findings to a broader population of cancer patients undergoing radiation therapy. Secondly, the relatively short duration of the observation period, set at one year, may not provide a comprehensive understanding of the long-term effectiveness of the interventions. It is recommended to consider extending the observation period to assess the sustainability of the outcomes over a longer timeframe. Additionally, the study sample predominantly focuses on first-time breast cancer patients, which may limit the applicability of the results to patients with different cancer types. To enhance the external validity, future research should strive to include a more diverse and representative sample of patients. Finally, it is worth noting that the use of multimedia education, although shown to be more effective in promoting self-care among patients, may pose challenges in terms of reduced interaction time with nursing staff, potentially impacting the perceived personalization of the educational approach.

## Conclusions

Among breast cancer patients who received radiotherapy for the first time, we observed a higher level of engagement with multimedia teaching materials in terms of self-care comprehension, with 100% completion, compared to only 45% for the health education leaflets. Our results also indicated significant improvements in comprehension rates for both groups, although the patients who received multimedia teaching materials showed higher rates of improvement. Using multimedia tools has several advantages, including providing customized, step-by-step training based on individual educational needs and feedback. This can help patients adhere to medical instructions and improve their self-care performance. However, in terms of nursing staff satisfaction, those who implemented multimedia teaching materials reported lower satisfaction compared to those who used traditional health education leaflets. This suggests that when implementing multimedia materials for self-care education, it is important for the nursing staff to pay attention to communication with patients in order to maintain the overall quality of medical care.

## Data Availability

The authors declare follows the Nature Portfolio policies for the sharing of research materials.

## Abbreviations

QR: Quick response
CVI: Content validity index
